# Horace Wells: A Pioneer in Modern Anesthesia and Pain-Free Medical Practices

**DOI:** 10.7759/cureus.71575

**Published:** 2024-10-15

**Authors:** Laresh N Mistry, Nikita A Khachane, Pakhi P Shah, Mrunmayee Soman, Shreyas Neelkanthan, Ashwin M Jawdekar

**Affiliations:** 1 Department of Pediatric and Preventive Dentistry, Bharati Vidyapeeth (Deemed to Be University) Dental College and Hospital, Navi Mumbai, IND

**Keywords:** anesthesia, conscious sedation, inhalation, nitrous oxide, pediatric dentistry, tooth extraction

## Abstract

Horace Wells, a pioneering figure in the field of anesthesia, was born on January 21, 1815, in Hartford, Vermont. He is widely recognized for his groundbreaking discovery of the anesthetic properties of nitrous oxide, a finding that revolutionized pain management in both medical and dental procedures. Wells’ contribution marked a pivotal moment in the history of surgery, transforming it from a traumatic, painful ordeal to a more humane and tolerable practice.

Wells initially trained as a dentist and began his career in Hartford, USA, where he developed a reputation for his skill and innovation. His interest in finding a solution to the extreme pain patients experienced during dental work led him to experiment with nitrous oxide, also known as "laughing gas." In 1844, after observing its recreational use, Wells hypothesized that nitrous oxide could be used to alleviate pain during surgical procedures. To test his theory, Wells arranged a public demonstration in which he had his own tooth extracted while under the influence of nitrous oxide. The experiment was a success, proving the anesthetic potential of the gas.

Although Wells’ initial public demonstration in Boston met with skepticism and failure, his work laid the foundation for the development of anesthesiology. Over time, the medical community recognized the significance of his discovery, which opened the door to further advancements in anesthesia, including the use of ether and chloroform.

Wells’ contributions, particularly to dental surgery and anesthesia, continue to be celebrated. He is remembered as one of the key figures who helped establish the practice of pain-free surgery, changing the course of both medical and dental fields forever. Despite facing early criticism, Wells’ discovery of nitrous oxide remains a cornerstone of modern anesthesia.

## Introduction and background

Anesthesia, when discussed, brings certain names to the surface who are worthy of acknowledgment. Horace Wells is an eminent personality who made a remarkable contribution to the concept of pain-free surgery by studying the effects of nitrous oxide. Regarding it, he stated, “Let it be as free as air we breathe.”

In 1815, Horace Wells was born on 21st January in Hartford, USA, Vermont as the eldest son of Horace and Betsey Heath Wells followed by his brother Charles (1817) and sister Mary (1819) [1]. His grandfather, Captain Hezekiah Wells served in American Revolution. His grandmother, Sarah Trumbull was associated with Jonathan Trumbull who was the Governor of Connecticut during the American Revolution. His father was well off and possessed wide land near the Connecticut River [2]. During his first 12 years of life, he attended some of the finest schools such as a private boys’ school with Mr. Ballard in Hopkinton, New Hampshire, and some academies in Amherst, Massachusetts [2-4]. His parents were brilliant and taught good values and virtues and provided a good environment for mental growth to each of their children. During his formative years, Well showed a demonstrative and inventive ability, which led to him becoming an inventor in adult life [4]. After the competition of his early education, he was offered a job to teach writing for several schools. At one point in time, he considered ministry as his career due to the intense effect of his father’s death on his life [2]. Yet, this idea was short-lived, and he later considered dentistry as his career for reasons yet unknown. Maybe it was the influence of voyaging dentists who stayed at his father’s house, which was used as a hotel for passersby of Connecticut Valley [2].

Thus, to pursue dentistry, he traveled to Boston at the age of 19, where he started an apprenticeship in 1834 for two years under the direction of a recognized practitioner as the first dental school, The Baltimore College of Dental Surgery, established in the year 1834 [2,4]. Due to the privileges of his parents, it is assumed that he did his apprenticeship under Dr. N. C. Keep who has received immense recognition for his expertise in mechanical dental art. He further went on to be named Dean of the recently started Harvard Dental School in the year 1868 at the age of 68 [2,4].

After completing his traineeship, he then relocated back to Hartford, where he successfully established his dental practice by printing a news article in the Connecticut Courant where he stated the facility he provided as a trained dentist for his patients who considered conservation of natural teeth using gold restorations [2,3]. He used to invent his own instruments which he used in his own practice which made him a popular dentist among the people and elites of the city. At a local exhibition, he won an award from the Massachusetts Mechanical Association for the instrument case which he designed and built himself. In 1836, he told his family about his profits which were from $5 to $20 per day. By 1838, he reported profits of up to $100 per week [4]. He shifted to as many as six different places between 1836 and 1845 [2,4]. He faced a few problems while shifting to different places as the instruments used at that time were not electricity-driven. He was even satisfied with just natural light as his light source while doing dental procedures. He carried his own instruments, such as forceps, handheld burs, and drills, teeth, and restorative materials such as gold with him [4].

## Review

In the year 1838, he wrote a small book named “An Essay on Teeth” that described the development of teeth, oral diseases, and treatments for the same [2,4]. He was an advocate of a well-balanced diet and oral hygiene and gave theories of infection that were advanced for that period. He was a very strong advocator of preventive practices due to which an entire chapter was devoted to the same in his book. He recommended the use of a brush for teeth cleaning and quoted “Those teeth which are frequently cleansed with a brush seldom or never decay" [4]. He was very well-reputed for treating children. He understood the harmful effects of sugar and wrote, “There is nothing more destructive to teeth than a compound sold at nearly every corner of the street, under the name of candy.” He was also a pioneer in identifying the need to preserve primary dentition and stated, “primary teeth be allowed to remain until they are ready to fall out of themselves unless they become too troublesome to be endured.” Wells also understood the most important form of dental treatment to be filling carious teeth and wrote, “However simple the operation of filling the teeth may appear, it is, in reality, the most complicated, as well as the most important branch of the profession" [4].

In 1838, he married Miss Elizabeth Wells and became a father on August 26, 1839, to his only son Charles Wells [1,2]. During the many years of his practice in Hartford, he instructed many students about the art of dentistry [5]. Among them, significant students were Dr. William T. G. Morton who is recognized for his experiments on ether as a potent anesthetic agent, and Dr. John M. Riggs who was well-known for “Riggs Disease” [2,5]. He started an office in partnership with Dr. Morton in Boston; however, that did not last long [2] After a short period of time, he was awarded a certificate from the renowned chemist and geologist Dr. Charles Jackson (his medical school guide) for his invention of gold solder [2]. During his time, life expectancy was around 35 years and smallpox was the only communicable disease that was rapidly spreading. Hospitals were known as the “House of Death” and major surgeries were considered a “virtual death sentence” [4].

A huge part of Wells’ dental practice was the extraction of teeth which was evidenced by the huge number of forceps in his inventory and his day-book. He was distressed about the pain and discomfort of the patient after extractions. Hence, he was in search of an agent that would minimize the pain and discomfort of his patients after tooth extraction. In 1840, in a discussion with Linus P. Brockett, a physician of Hartford he was “deeply impressed with the idea that some discovery would yet be made by which dental and other operations might be performed without pain” [4].

In 1844, on December 10, a Grand Exhibition on the demonstration of nitrous oxide was organized by Dr. Gardener Quincy Colton as, “A Grand Exhibition of the Effects Produced by Inhaling Nitrous Oxide, Exhilarating, or Laughing Gas” [6] which he organized for public entertainment [3]. Wells and his wife both attended the event [6]. The audience members were invited to inhale the gas to experience the effects as part of the exhibition for which Wells volunteered on which his wife quoted that he “made a spectacle of himself” [4,5]. The next person to volunteer was Samuel Cooley who was sitting next to Wells [7]. Cooley stated that “when he was administered the gas, he was immediately intoxicated, became giddy, and began jumping about the stage” [8] which led to him getting injured by banging his leg on the wooden bench. After the gas effects had worn off, he was unable to recall the experience and he “found that the skin (on his knees) was severely abraised and broken [8] and when he was asked about his pain perception, he denied having experienced any kind of pain while under the influence of the nitrous oxide gas [4]. According to Colton, this was noticed by Wells [7]. Colley stated that “if a person could be restrained…he could undergo a severe surgical operation without feeling any pain at the time.” This intrigued Wells, and he responded “that he believed that a person could have a tooth extracted while under (nitrous oxide) influence and not feel pain [8]. He then asked Dr. Colton if he was willing to participate in his own wisdom tooth extraction by his colleague Dr. Riggs [8-12].

On the very next day, that is December 11, 1844, a bag of nitrous oxide was brought by Colton, and in the presence of Riggs, Wells inhaled the gas from the paper bag [11,12]. While Wells was under the influence of the gas, Dr. Riggs extracted Wells’ wisdom tooth [4]. Wells showed no discomfort in the entire duration of the procedure [9,11]. When he was asked about his pain perception, he responded, “It is the greatest discovery ever made! I didn’t feel it so much as the prick of a pin!” [12] A new era in tooth-pulling! [11]. At the request of Wells, Colton taught him the way to prepare the nitrous oxide gas. During the following weeks, Wells and Riggs used nitrous oxide gas during teeth extractions as an anesthetic. Wells used the gas on as many as 12 patients [9] and Riggs once as a part of a procedure in which in one sitting six teeth were extracted [13].

Wells traveled to Boston to share his findings. With the help of a former student and partner, Dr. Morton organized a session to illustrate the experiment on nitrous oxide sedation in the presence of Dr. John Collins Warren the Chief of Surgery at Massachusetts General Hospital [13-16]. After his lecture, he waited for days for the actual demonstration which was originally scheduled to provide anesthesia for an amputation [9]. Alternatively, Wells agreed to administer nitrous oxide for the extraction of a tooth from the patient. Wells however retrieved the bag even before the gas had started showing its effects and continued with the extraction to which the patient cried out in pain [5]. Later, the patient however admitted that he did not actually feel the removal of the tooth, but the demonstration was still said to be “an imposition” [10] and a “humbug affair” [6], and Wells was tagged a “charlatan” and a “fake” [5].

Wells on his return to Hartford was dejected and had a mental breakdown which led to him ceasing his dentistry practice by way of an announcement of closing his practice and referring all his patients to Dr. Riggs on April 7, 1845, in the Hartford Courant by stating “excitement of this adventure (Boston) brought on an illness from which I did not recover for many months, being thus obliged to relinquish entirely my professional business” [4,17]. Later he continued the use of nitrous oxide sedation for dental procedures and even requested other dentists to use it along with training other dentists who were unaware of the use of the gas. He even facilitated surgeons with anesthesia for certain surgeries such as amputations and tumor removals between 1846 and 1848 [2,4]. Additionally, he dived into other interests such as natural history for which he arranged an exhibition in Hartford in 1845 as well as the design and manufacture of a new type of shower bath for which he stopped practice again [4].

In 1846, on October 10, the effects of ether were illustrated by Dr. Morton which led to the naming of that amphitheater as “Ether Dome” which was published in *Boston Medical and Surgical Journal* in November [18]. Wells was happy about the achievement of his student and partner Dr. Morton yet was dejected when Dr. Morton proclaimed himself to be the pioneer of anesthesia [4] for which his wife stated that this led to the “gas war” [4]. Wells published in December 1844 the events to establish a claim for him being the discoverer of anesthesia [19] for which he petitioned the Academie Royale de Medicine (Parisian Medical Society) in 1847 [6]. On his return back to his hometown, he continued his self-experiments with nitrous oxide anesthesia with the addition of ether and chloroform [16]. However, while performing self-experiments he became addicted to chloroform leading to mental disturbances [4]. Once, while under the intoxication of chloroform, he threw sulphuric acid on whom he addressed as “abandoned females.” For this heinous act, he was held guilty and imprisoned in Tombs Prison on January 21, 1848 [2,6]. The next day while under surveillance he went home to get a few of his belongings which included some chloroform and a razor that he smuggled into prison [2]. On January 24, 1848, Wells committed suicide by inhaling the smuggled chloroform and slashing his femoral artery [6].

However, 12 days before his death Wells was voted to henceforth be recognized as “the first person, who first discovered and performed surgical operations without pain…and to the last day of time must suffering humanity bless his name” and was “due all the honor of having successfully discovered and applied the use of vapors or gases whereby surgical operations could be performed without pain.” Yet this arrived in New York past his death [20].

Wells died merely at the age of 33, without the knowledge of the honor that he was awarded for his work, the title he had achieved as “Father of Anaesthesia,” and even the recognition he got from the Parisian Medical Society which was given just before his death [1]. Every physician in Hartford, Connecticut testified Wells was the primary discoverer of anesthesia by signing a document [1].

American Dental Association in 1864, released a recognition statement stating “that to Horace Wells of Hartford, Connecticut belongs the credit and honor of the introduction of anesthesia in the USA” [1]. The American Medical Association in May 1870 at its yearly session in Washington DC agreed “that the honor of the discovery of practical anesthesia is due to the late Horace Wells of Connecticut” [1]. Baltimore College of Dentistry posthumously bestowed the Doctor of Dental Surgery degree in the year 1994 [1]. On the 50th anniversary of Wells's discovery, in the year 1894, an organization of dentists and physicians was formed in Hartford, Connecticut which was named “The Horace Wells Club” [1]. They provide the facility of scholarships to Connecticut dental students who show significant accomplishment in the field of sedation and annually award an individual with significant accomplishment in the same field. They look after the statue of Horace Wells in Hartford’s Bushnell Park. They arrange fundraisers in honor of Horace Wells and the raised funds are used to maintain and restore Wells's tomb in Cedar Hill Cemetery where he was buried after his death as well as contribute to the local suicide prevention organization [1].

**Figure 1:**
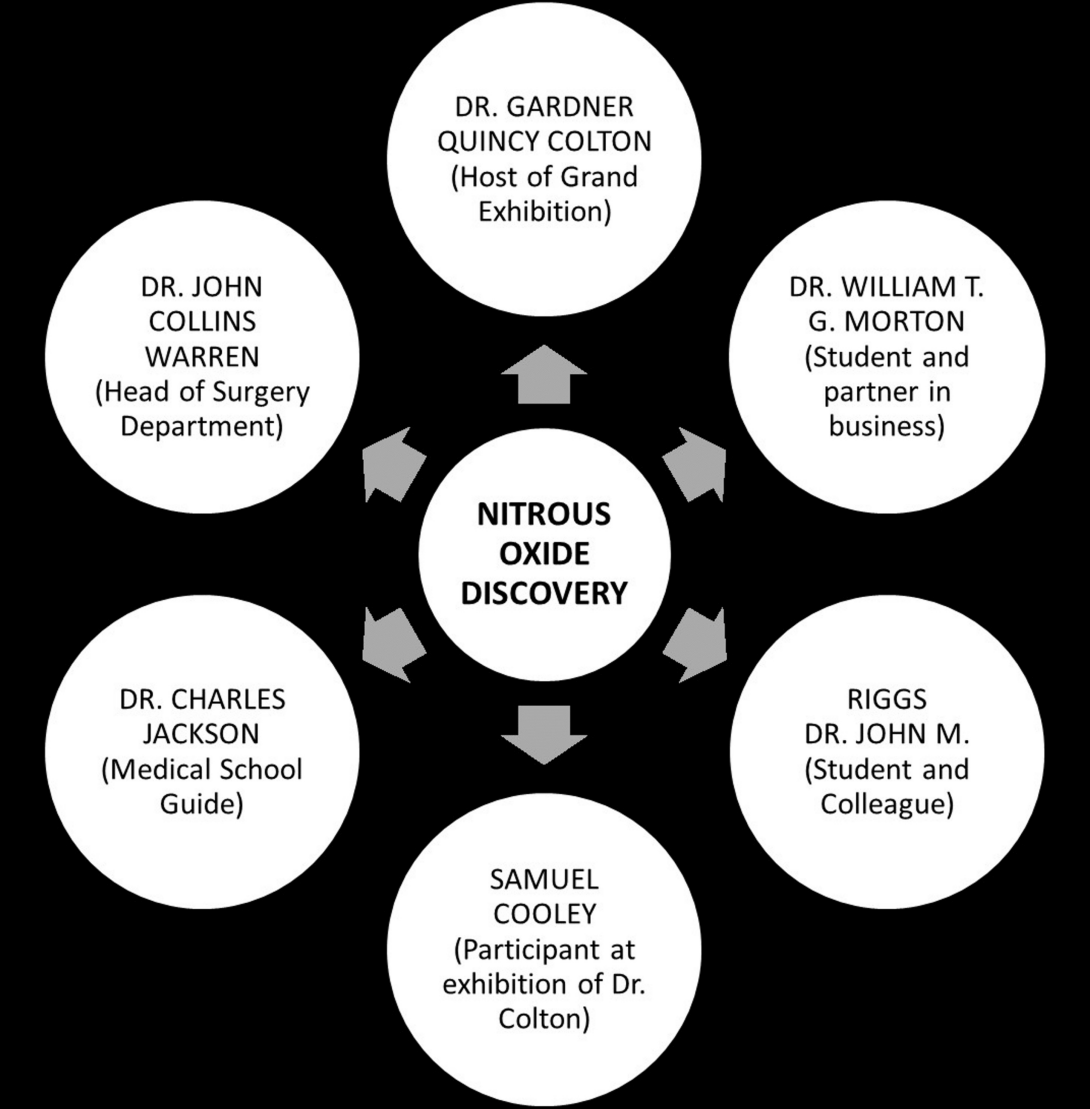
The discovery of nitrous oxide for anesthesia by Dr. Horace Wells

A timeline of Dr. Horace Wells's discovery of nitrous oxide and the honors he achieved, also highlighting his notable contribution to anesthesia (Figure 1).

**Figure 2:**
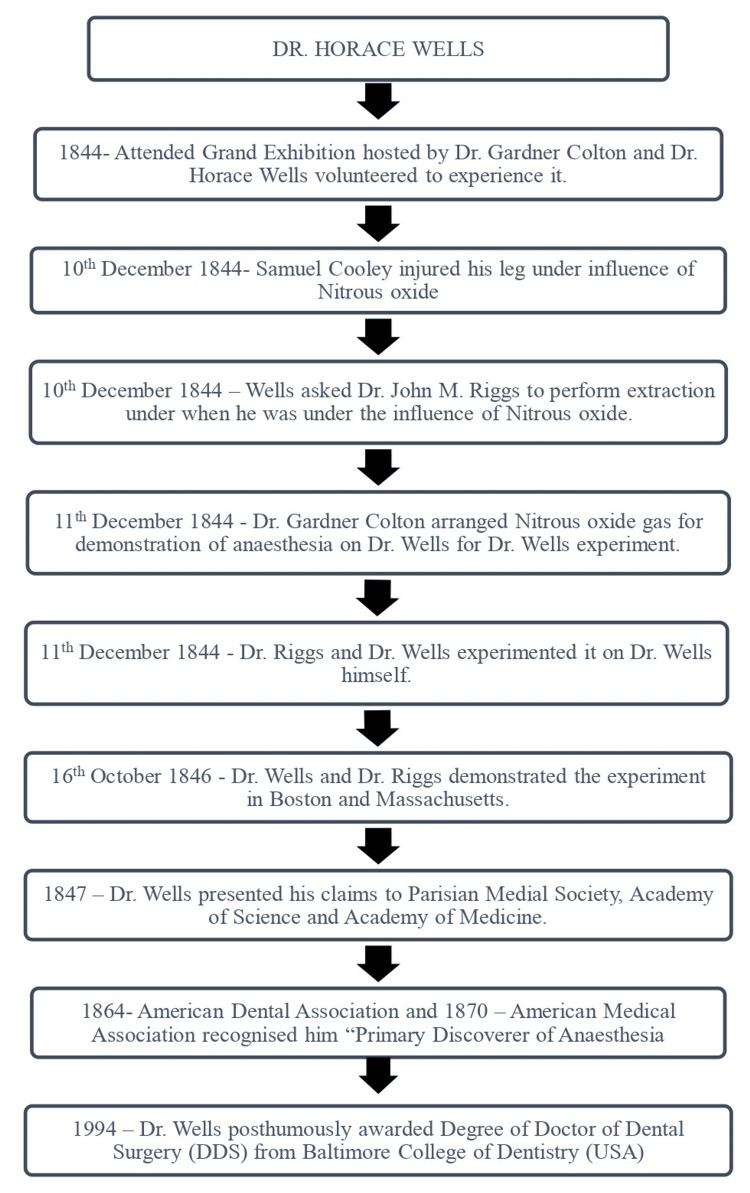
Recognition of Dr. Horace Wells

Dr. Horace Wells's groundbreaking work in anesthesia and his journey attending the exhibition arranged by Dr. Colton to being honored with a Doctor of Dental Surgery degree posthumously (Figure 2).

Comparison with its rivals

Ether gas as an anesthetic agent was discovered by Dr. William Morton. When it was demonstrated in front of people in an amphitheater in Massachusetts General Hospital which was further called an “ether dome,” it showed lesser success just like the nitrous oxide gas demonstration done in the same hospital. Ether had a pungent odor, a short duration of action, and was flammable. Being flammable, it could not be used for cautery procedures. Thus, it was not accepted by people for anesthesia.

Chloroform was used to its full potential by Dr. James Simpson (English obstetrician) in 1847. Chloroform had a shorter time of onset of action and a lesser concentration of chloroform was needed to produce an anesthetic effect as compared to the concentration of ether required for producing the same amount of anesthetic effect. Even though it is a more powerful agent as compared to ether, more deaths were reported with the use of the same.

Nitrous oxide has been known as laughing gas. Its potential use in dental operations was discovered by Dr. Horace Wells while its discovery as a gas was by Sir Humphrey Davy in 1776. It achieves its excitatory effects even if moderately inhaled. When the gas is in a pure state, it is colorless, odorless, and has a sweet taste. Nitrous oxide has a longer duration of action and fewer side effects compared with ether and chloroform [20].

In early times nitrous oxide was not completely used for anesthesia. It had its rivals in the form of ether (discovered by Dr. William Morton) and chloroform (discovered by Dr. James Simpson) which were used. The use of ether and chloroform was then reduced dramatically due to their adverse effects. Soon after that, nitrous oxide use was increased after the realization of its potency and useful properties. In modern dentistry, nitrous oxide has helped in achieving good treatment outcomes. In pediatric dentistry, it serves as a cheaper alternative to general anesthesia for the easy handling of an anxious child as well as intellectually disabled patients. A study by Galeotti et al. involving 688 pediatric patients, aged 4 to 17 years showed a successive rate of 86.3% of nitrous oxide conscious sedation [21]. A study by Perez et al. on evidence of equimolar nitrous oxide effectiveness reports high satisfaction and success rates for dental procedures [22].

## Conclusions

At the beginning of the 19th century, hospitals were often referred to as an abode of demise, and surgeries were regarded as nearly synonymous with a death sentence due to the unbearable pain involved. Dr. Horace Wells discovered nitrous oxide as an anesthetic agent, which brought an emergent change in medical science. This breakthrough laid the groundwork for the development of anesthesia, leading to further discoveries such as ether and chloroform. As a result, anesthesiology emerged as a distinct branch of medicine, owing its origins to the contribution of a dentist.

Nitrous oxide, initially introduced by Wells, has since played a crucial role in modern dentistry, especially in pediatric care. Its use in pediatric dentistry has become increasingly important, offering effective pain management and reducing anxiety during procedures. This pivotal discovery by a dentist not only transformed surgical practices but also continues to shape the evolution of the medical and dental fields.

## References

[1] Archer W (1939). Chronological History of Horace Wells Discoverer of Anesthesia. Chronological History of Horace Wells Discoverer of Anesthesia.

[2] Archer W (1944). Life and Letters of Horace Wells Discoverer of Anesthesia. Journal of American College of Dentist, Vol II, No. 2, June.

[3] Westhorpe R (1996). Horace Wells (1815-1848). Anaesth Intensive Care.

[4] Jacobson H (1995). Horace Wells: discoverer of anesthesia. Anesth Prog.

[5] Clark M, Brunick A (2014). Handbook of Nitrous Oxide and Oxygen Sedation.

[6] Finder S (1995). Lessons from history: Horace Wells and the moral features of clinical contexts. Anesth Prog.

[7] Colton GQ (1886). Anaesthesia. Who Made and Developed This Great Discovery? A Statement. New York, A.G. Sher- wood.

[8] Cooley SA (1858). Extract from the deposition of Samuel A. Cooley, of Hartford, Connecticut. An Examination of the Question of Anaesthesia, Arising on the Memorial of Charles Thomas Wells, Presented to the United States Senate, Second Session, Thirty-Second Congress, and Referred to a Select Committee, of Which the Hon. Isaac P. Walker Is Chairman.

[9] Wells H (1847). A History of the Discovery of the Application of Nitrous Oxide Gas, Ether, and Other Vapors, to Surgical Operations. Hartford, CT, J. Gaylord Wells,1847.

[10] Wells E (1858). Extract from the deposition of Elizabeth Wells, of Hartford, Connecticut. An Examination of the Question of Anaesthesia, Arising on the Memorial of Charles Thomas Wells, Presented to the United States Senate, Second Session, Thirty-Second Congress, and Referred to a Select Committee, of Which the Hon. Isaac P. Walker Is Chairman.

[11] Riggs JM (1858). Extract from the deposition of John M. Riggs, dentist of Hartford, Connecticut. An Examination of the Question of Anaesthesia, Arising on the Memorial of Charles Thomas Wells, Presented to the United States Senate, Second Session, Thirty-Second Congress, and Referred to a Select Committee, of Which the Hon. Isaac P. Walker Is Chairman.

[12] Colton GQ (1858). Deposition of G. Q. Colton, of the city of New York. An Examination of the Question of Anaesthesia, Arising on the Memorial of Charles Thomas Wells, Presented to the United States Senate, Second Session, Thirty- Second Congress, and Referred to a Select Committee, of Which the Hon. Isaac P. Walker Is Chairman.

[13] Davison MHA (1957). The evolution of anaesthesia. Br J Anaesth.

[14] Finder SG, DiPersio DM (1996). Relationships with patients and physicians. Ethical Issues in Pharmacy.

[15] Robinson V (1946). Victory Over Pain.

[16] Nuland SB (1983). The Origins of Anesthesia.

[17] (2024). Wells: classified advertisement. https://www.woodlibrarymuseum.org/wp-content/uploads/rare-books/S_ABYZ.pdf.

[18] Bigelow HJ (1846). Insensibility during surgical operations produced by inhalation. Boston Med Surg J.

[19] Brewster CS (1946). Letter to Wells. Victory Over Pain.

[20] Bruce W (1869). Anæsthetics for dental operations: ether, chloroform and nitrous oxide. Am J Dent Sci.

[21] Galeotti A, Garret Bernardin A, D'Antò V (2016). Inhalation conscious sedation with nitrous oxide and oxygen as alternative to general anesthesia in precooperative, fearful, and disabled pediatric dental patients: a large survey on 688 working sessions. Biomed Res Int.

[22] Perez A, Gernandt S, Scolozzi P (2023). The use of equimolar mixtures of nitrous oxide and oxygen in oral surgery - a retrospective study of patients in a Swiss university hospital setting. J Clin Med.

